# A Synthetic Agonist to Vasoactive Intestinal Peptide Receptor-2 Induces Regulatory T Cell Neuroprotective Activities in Models of Parkinson’s Disease

**DOI:** 10.3389/fncel.2019.00421

**Published:** 2019-09-18

**Authors:** R. Lee Mosley, Yaman Lu, Katherine E. Olson, Jatin Machhi, Wenhui Yan, Krista L. Namminga, Jenell R. Smith, Scott J. Shandler, Howard E. Gendelman

**Affiliations:** ^1^Department of Pharmacology and Experimental Neuroscience, Center for Neurodegenerative Disorders, University of Nebraska Medical Center, Omaha, NE, United States; ^2^Longevity Biotech, Inc., Philadelphia, PA, United States

**Keywords:** neurodegeneration, Parkinson’s disease, vasoactive intestinal peptide, agonist, microglia, regulatory T cells, alpha-synuclein, 6-hydroxydopamine

## Abstract

A paradigm shift has emerged in Parkinson’s disease (PD) highlighting the prominent role of CD4^+^ Tregs in pathogenesis and treatment. Bench to bedside research, conducted by others and our own laboratories, advanced a neuroprotective role for Tregs making pharmacologic transformation of immediate need. Herein, a vasoactive intestinal peptide receptor-2 (VIPR2) peptide agonist, LBT-3627, was developed as a neuroprotectant for PD-associated dopaminergic neurodegeneration. Employing both 6-hydroxydopamine (6-OHDA) and α-synuclein (α-Syn) overexpression models in rats, the sequential administration of LBT-3627 increased Treg activity without altering cell numbers both in naïve animals and during progressive nigrostriatal degeneration. LBT-3627 administration was linked to reductions of inflammatory microglia, increased survival of dopaminergic neurons, and improved striatal densities. While α-Syn overexpression resulted in reduced Treg activity, LBT-3627 rescued these functional deficits. This occurred in a dose-dependent manner closely mimicking neuroprotection. Taken together, these data provide the basis for the use of VIPR2 agonists as potent therapeutic immune modulating agents to restore Treg activity, attenuate neuroinflammation, and interdict dopaminergic neurodegeneration in PD. The data underscore an important role of immunity in PD pathogenesis.

## Introduction

Parkinson’s disease (PD) is a progressive degenerative movement disorder characterized by loss of dopaminergic neurons in the substantia nigra pars compacta (SNpc) and their striatal connections. No therapies are available to date that alter disease progression. While the etiology of PD remains enigmatic, misfolded and nitro-oxidation modified alpha synuclein (α-Syn) produce neurotoxic protofibrils and form Lewy bodies. After extraneuronal release of protofibrils, microglial activation and inflammation ensue with consequent neuronal damage resulting in a cascade of misfolding self-proteins and chronic inflammation leading to progressive neurodegeneration.

In addition to the innate microglial neuroinflammation linked to neurodegeneration, peripheral adaptive immunity is recognized to influence neurodegeneration and, as such, opens new opportunities for therapy (Ha et al., 2012; Olson et al., 2015, 2016; Deng and Jin, 2017; Lee et al., 2017; Gelders et al., 2018). This is highlighted by the key roles that CD4^+^ T cell subsets play in the pathobiology of neurodegenerative diseases. In support of the linkages between immunity and PD, genetic studies have recently identified links between MHC II and disease (Kannarkat et al., 2015). Indeed, the delicate balance between effector T cells (Teffs) and Tregs affect neurodestructive and neuroprotective outcomes for neurodegenerative activities (Reynolds et al., 2010; Mosley et al., 2012; Anderson et al., 2014; Gendelman and Mosley, 2015; Mosley and Gendelman, 2017). Thus, either a minimum frequency or an appropriate function of peripheral Tregs appear to be vital for central nervous system (CNS) homeostasis. The number of Tregs or their activity are diminished in PD, stroke, and amyotrophic lateral sclerosis (ALS), and as a consequence lead to changes in the diseased-brain microenvironment with concurrent oxidative stress, inflammation, and protein misfolding; all serve to augment or speed neurodegenerative processes (Beers et al., 2011; Rentzos et al., 2012; Saunders et al., 2012; Henkel et al., 2013; Hu et al., 2014; Chen et al., 2015; Gendelman et al., 2017; Duffy et al., 2018). As a result, therapeutic strategies have emerged employing classes of immunomodulatory agents to promote T cell differentiation or to increase their baseline activity that lead to attenuation in both inflammation and oxidative stress that emerge during disease (ClinicalTrials.gov Identifier: NCT03790670) (Romero-Ramos et al., 2014; Gendelman et al., 2017; Kustrimovic et al., 2018; Solleiro-Villavicencio and Rivas-Arancibia, 2018).

One Treg-promoting agent is vasoactive intestinal peptide (VIP), a 28-amino acid polypeptide with pleiotropic activities that also serves as a neurotransmitter for central and autonomic nervous systems (Said, 2007). Pleiotropic activities for VIP include effects on vasodilation, secretion, circadian rhythm, memory recall, and inflammation through activation of specific class B GPCRs, namely VIPR1 (VPAC1) and VIPR2 (VPAC2), as well as with the lower affinity pituitary adenylate cyclase activating polypeptide 1 receptor type 1 (ADCYAP1R1 or PAC1) (Said, 2007; Vosko et al., 2007; Gomariz et al., 2010; Waschek, 2013; Fan et al., 2015; Kamigaki and Dan, 2017). VIP profoundly affects innate and adaptive immunity (Delgado and Ganea, 2013), and in models of autoimmunity exerts immune modulatory activities. VIP treatment strategies for rheumatoid arthritis, type I diabetes, Sjögren’s disease, inflammatory bowel disease, experimental autoimmune encephalomyelitis, and autoimmune uveitis are in development (Delgado et al., 2001; Juarranz et al., 2005; Fernandez-Martin et al., 2006; Deng et al., 2010; Jimeno et al., 2010; Ganea et al., 2015). VIP effects on inflammatory-mediated autoimmune disorders are elicited, in large measure, by induction of Tregs through tolerogenic DCs (Chorny et al., 2005; Maldonado and von Andrian, 2010). In PD, works from other and our laboratories have shown that VIP elicits significant dopaminergic neuroprotective responses in PD models (Delgado and Ganea, 2003; Reynolds et al., 2010; Olson et al., 2015). VIP is up-regulated in neuroimmune cells after injury (Kim et al., 2000; Sandgren et al., 2003; Nishimoto et al., 2011). VIP also affects inflammatory response cascades, including mononuclear phagocytes (MP; macrophage and microglia) and T cells by inhibiting production or release of inflammatory cytokines such as tumor necrosis factor-alpha (TNF-α) and interferon-gamma (IFN-γ) (Gonzalez-Rey and Delgado, 2005, 2008; Delgado et al., 2008; Waschek, 2013; Higyno et al., 2015). Furthermore, a critical aspect of the underlying biology is that VIP has been shown to rebalance the polarization of T cell responses from type-1 T helper cell (Th1) and type-17 Th cell (Th17) toward a type-2 Th cell (Th2) phenotype (Jimeno et al., 2012; Tan et al., 2015; Villanueva-Romero et al., 2018). Additionally, studies have demonstrated that VIP elicits both natural and inducible subsets of Tregs (Delgado et al., 2005; Fernandez-Martin et al., 2006; Szema et al., 2011). The neuroprotective and immunomodulatory actions of VIP, and the related pituitary adenylate cyclase activating polypeptide (PACAP), support the idea for the development of VIP- and PACAP-activated receptors as therapeutic targets for neurodegenerative and neuroinflammatory diseases (Tan and Waschek, 2011; Waschek, 2013; Olson et al., 2015, 2016).

We synthesized two novel agonists that discriminate VIPR1 and VIPR2 and exhibit increased systemic stability with greater than 20-fold longer half-lives compared to native VIP (Olson et al., 2015). Treatment of naïve animals with the VIPR2 agonist, LBT-3627, increased Treg activity without expanding Treg numbers. We previously compared the neuroprotective capabilities of these selective peptides in an acute 1-methyl-4-phenyl-1,2,3,6-tetrahydropyridine (MPTP) model of dopaminergic neurodegeneration. Pre-treatment with the VIPR2 agonist, LBT-3627, provided robust and greater dopaminergic neuronal protection compared to pre-treatment with VIPR1 agonist or native VIP (Olson et al., 2015, 2016). Additionally, adoptive transfer of Treg-mediated activity from VIPR2 agonist- or VIP-treated animals decreased reactive microglia numbers and pro-inflammatory cytokine production by microglia (Reynolds et al., 2010; Olson et al., 2015). Moreover, that co-transfer of VIP-induced Tregs with nitrated-α-Syn-specific Th17 Teffs to MPTP intoxicated recipient mice provided greater levels of neuroprotection than non-induced Tregs, and that adoptive transfer of LBT-3627-induced Tregs afforded greater neuroprotection than VIP-induced Tregs, highlight the potential importance of VIPR2 agonists as a putative treatment for neurodegenerative disorders.

Although LBT-3627 induced Treg-mediated neuroprotection in MPTP intoxicated mice, whether VIPR2 agonist induction of Tregs is conserved and protective in diverse models of PD is not known. To these ends, the VIPR2 agonist, LBT-3627 was tested for its abilities to augment Treg numbers and function, and to elicit neuroprotective activities in two models of nigrostriatal neurodegeneration. Stereotactically delivered 6-hydroxydopamine (6-OHDA) was used as a neurotoxic alternative to MPTP as no known immune toxic effects have been reported, while stereotactic overexpression of human α-Syn from adeno-association human α-Syn (AAV-α-Syn) constructs was also used as the slower progression is considered a better model to capture the pathobiology of PD (St Martin et al., 2007; Theodore et al., 2008; Torres et al., 2011; Decressac et al., 2012). Additionally, the 6-OHDA model was included as considerable evidence showed VIP provided protective effects as demonstrated by diminished oxidative stress and apoptosis, and improvements in measures of dopaminergic neuron survival, spine densities for medium spiny neurons, neurotransmitter levels, synaptic plasticity, rotational behavior, and striatal astrocyte activity (Tuncel et al., 2005, 2012; Korkmaz et al., 2010, 2012; Yelkenli et al., 2016; Korkmaz and Tuncel, 2018). In these studies, both immune function and neuroprotection by LBT-3627 were evaluated in both models at multiple dose levels to determine parallel model effects. The results indicated that LBT-3627 diminished the number of reactive microglia at all doses tested. Moreover, we demonstrated that this VIPR2 agonist enhances Treg function peripherally, which leads to attenuation of microglial inflammatory responses and increased dopaminergic neuroprotection at dose levels that can be translated for human use. Lastly, due to the historical nature of VIP and the role of VIP2R in vasodilation (Koga et al., 2014), initial pre-clinical testing of LBT-3627 was performed for heart rate, pulse, and systolic blood pressure. All readings recorded were within average values. Taken together, we provide strong translational merit toward moving forward a unique pharmaceutical agent for PD neuroprotection.

## Materials and Methods

### Animals

Male 7-week old Sprague Dawley and Lewis rats were ordered from SASCO and Charles River Laboratories, respectively. Typical weights were 180–200 g at the time of surgery or regulatory T cell (Treg) functional studies. All rodent procedures were performed with approval of the UNMC Institutional Animal Care and Use Committee and in accordance with NIH Guide for the Care and Use of Laboratory Animals.

### LBT-3627 Treatment and Stereotactic Injections of 6-OHDA and AAV Vectors

Rats were treated initially with 5 daily s.c. doses of LBT-3627 at 0.06, 0.2, 0.6, 2.0, or 6.0 mg/kg/100 μl dose. Animals treated with an equal volume of PBS vehicle served as negative controls. Immediately after delivery of AAV vectors or 6-OHDA, rats began a regimen of LBT-3627 or vehicle for 5 days and every other day thereafter. For stereotaxic delivery, rats were anesthetized with 2% isoflurane in oxygen and placed in a stereotaxic device (Leica Biosystems Inc., Buffalo Grove, IL, United States). For each rat, the scalp was retracted to expose the skull and a 1–2 mm hole was drilled in the skull. Injections were accomplished using sterile Hamilton syringes (model 8100) affixed with 26-gauge needles and delivered by with syringe pump (LEGATO 210, cat 78-8212, KD Scientific, Holliston, MA, United States). For α-Syn overexpression studies, AAV2/1-α-SYN-IRES-eGFP-WPRE (AAV-α-Syn) (cat Standaert-5713) or control AAV2/1-IRES-eGFP-WPRE (AAV-GFP) (cat Standaert-5712) were obtained from the University of Iowa Vector Core (Iowa City, IA, United States) with kind permission from Dr. David G. Standaert (Department of Neurology, University of Alabama-Birmingham, Birmingham, AL, United States) (St Martin et al., 2007; Theodore et al., 2008). For these studies, 3 × 10^9^ genomic copies of AAV-vectors were delivered in 3 μl of PBS to the left hemisphere above the substantia nigra at coordinates relative to the bregma, AP, −5.3 mm; ML, −2.0 mm; and −7.5 mm according to the stereotaxic atlas (Paxinos and Watson, 1986; Decressac et al., 2012). For studies using 6-OHDA, rats received 10 μg of 6-OHDA in 5 μl PBS delivered to the right hemisphere above the medial forebrain bundle at coordinates AP, −4.5 mm; ML, 1.5 mm; DV, −7.3 mm, and relative to the bregma (Paxinos and Watson, 1986; Torres et al., 2011).

### Immunohistochemistry and Stereological Analysis

Following administration of terminal pentobarbital anesthesia, rats were transcardially perfused with DPBS followed by 4% paraformaldehyde in DPBS. The cohort that received 6-OHDA injections were euthanized on day 14, while the cohort that received AAV-only injections were euthanized on day 28. Brains were obtained and cryosectioned through the ventral midbrain and striatum. Frozen midbrain sections (30 μm) were collected in PBS as free-floating sections, and every 8th section was immunostained for 48 h as free-floating sections for tyrosine hydroxylase (TH) (anti-TH, 1:2000, EMD Millipore, Burlington, MA, United States) (Benner et al., 2008). Sections were washed and reacted with biotinylated secondary antibody for 1 h and washed. To visualize antibody-labeled tissues, sections were incubated in streptavidin-HRP solution (ABC Elite Vector Kit, Vector Laboratories, Burlingame, CA, United States) and color was developed using a glucose/glucose oxidase/H_2_O_2_ generation system and diaminobenzidine (DAB) chromogen (Sigma-Aldrich). Sections were mounted to microscope slides, counterstained for Nissl substance, dried, coated with mounting medium (Cytoseal 60, Thermo Fisher Scientific), and covered with a coverslip. To determine the microglia reactivity, midbrain sections were immunostained as free-floating sections for ionized calcium binding adaptor molecule 1 (Iba1) (anti-Iba1, 1:1000, Wako, Richmond, VA, United States) and biotinylated secondary antibody, and were visualized as previously described. The slides were coded by an investigator not familiar with treatment regimens and were assessed by blinded investigators. Total numbers of reactive Iba1^+^ microglia/mm^2^, TH^+^Nissl^+^ (dopaminergic neurons), and TH^–^Nissl^+^ (non-dopaminergic) neurons in the substantia nigra were determined by stereological analyses using the Fractionator probe from StereoInvestigator software (MBF Bioscience, Williston, VT, United States) interfaced with an Eclipse 90i microscope (Nikon, Melville, NY, United States). To sample through the substantia nigra using every 8th section required 10 sections/rat for enumeration of total nigral neurons and microglia density. The densities of TH^+^ expression for striatal termini were determined by digital densitometry using image J software (National Institutes of Health, Bethesda, MD, United States) (Benner et al., 2008). Briefly for each rat, optical densities from 6 striatal sections stained for expression of TH were obtained from bit map probes of standardized areas, and background densities were determined from non-striatal areas. Striatal TH densities were determined for each section by subtracting the densities of background from TH^+^ areas and the mean of the 6 sections served as the striatal TH density/animal.

### Treg Function Assessment

**FIGURE 1:**
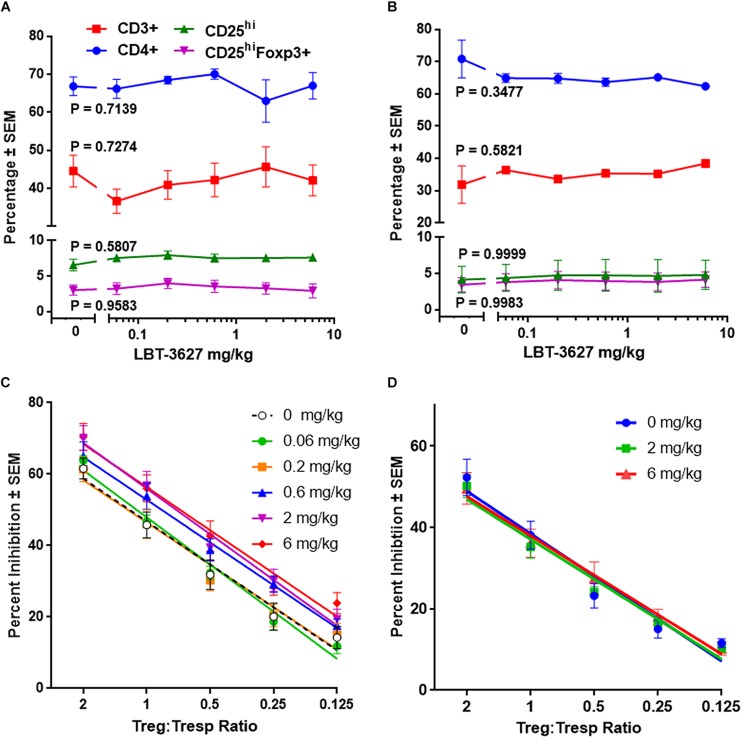
Multiple doses of VIPR2 agonist augments Treg activity. Naïve Lewis rats were administered 5 daily s.c. injections of PBS vehicle (designated 0) or LBT-3627 at doses of 0.06, 0.2, 0.6, 2.0, or 6.0 mg/kg (*n* = 5 rats/group). One day after last injection, cells from peripheral blood **(A)** and spleens **(B)** were assessed for T cell and Treg frequencies. Percentages of cell populations included CD3^+^ T cells among lymphocytes (red circles), CD4^+^ T cells among CD3^+^ cells (blue squares), CD25^hi^ T cells among CD3^+^CD4^+^ cells (green triangles), and CD25^hi^Foxp3^+^ among CD3^+^CD4^+^ cells (purple inverted triangles). Mean percentages and SEMs were determined from 5 rats/group and significant differences between dosages were assessed for each population by one-way ANOVA. *P* values for each T cell population are denoted next to the concentration curve. **(C)** Spleen cells obtained from the above multiple-dosed animals **(B)** or from rats receiving a single s.c. dose of LBT-3627 at 2.0 or 6.0 mg/kg (*n* = 5 rats/group) **(D)** were enriched for CD3^+^CD4^+^CD25^hi^ Tregs and assessed for Treg function. Percent Treg-mediated suppression was determined by flow cytometric analysis. Significant differences were determined from linear regression analysis with 5 rats/group and triplicate assays for each rat. Regression analysis for all lines indicated that *R*^2^ > 0.96 with *P* values < 0.0054.

CD4^+^CD25^+^ and CD4^+^CD25^–^ T cells were isolated from rat spleens using CD4^+^ T Cell isolation kit (cat 19642) and PE selective kit (cat 18557) (StemCell, Vancouver, BC, Canada). Briefly, isolated spleen cells were collected and placed in a petri dish with 70 μm mesh cell strainer in 5 ml HBSS. A syringe plunger was used to gently mash tissue to obtain single cells. Red blood cells were removed with ACK lysis buffer (3 mL per spleen). Spleen cells were stained to obtain CD4^+^ cells according to the manufacturer’s protocol. CD4^+^ cells were stained with anti-CD25 PE (cat 55486, BD Biosciences, San Jose, CA, United States) for 20 min at a concentration of 0.75 μg/ml per 15 × 10^6^ cells and anti-PE-magnetic beads were used for positive selection of CD4^+^CD25^+^ T cells. These cells were greater than 90% Tregs as determined by expression of forkhead box P3 (Foxp3) by flow cytometric analysis (Figure 1B). The CD4^+^CD25^–^ T cell fraction was collected in the flow through and served as conventional responding T cells (Tresps) to be used in proliferation assays. Tresps were labeled with carboxyfluorescein succinimidyl ester (CFSE) (cat C34554, Thermo Fisher Scientific). Tregs were serially diluted by twofold dilutions in a 96 well U-bottom microtiter plate to contain 100, 50, 25, 12.5, and 6.25 × 10^3^ Tregs in 100 μl of media. To each well was added 100 μl of 50 × 10^3^ CFSE-stained Tresps to yield Treg:Tresp ratios of 2, 1, 0.5, 0.25, and 0.125:1, and Tresps in media served as controls. To each well was added 25 × 10^3^ rat T cell activating CD3/CD28 beads (1 bead: 1 Tresp) and cultures were incubated at 37°C in 5% CO_2_ for 72 h. The cells were fixed with 1% formaldehyde in PBS and analyzed by flow cytometric analysis. T cell activating CD3/CD28 beads were prepared with Dynabeads M-450 epoxy (cat 14011, Thermo Fisher Scientific) conjugated with anti-rat CD3 (cat 5012338, Thermo Fisher Scientific) and anti-rat CD28 (cat 5014270, Thermo Fisher Scientific) according to manufacturer’s protocol. For conjugation, the CD3:CD28 ratio was 1:1 and bead to antibody ratio was 1000 beads with 200 μg antibody (100 μg CD3 and 100 μg CD28). Coupled beads were stored at 2–8°C at a concentration of 4 × 10^7^ beads/ml in PBS, pH 7.4 with 0.1% bovine serum albumin (BSA) and were utilized over a 1 year period.

### Flow Cytometric Analysis

Blood was collected from the rat left atrium to determine Treg number. Blood (50 μl) was stained with a mixture of BV-421-anti-CD3 (2.5 μl, cat 563948, BD Bioscience), PerCP-eFluor 710-anti-CD4 (2 μl, cat 46-0040-82, eBioscience, Thermo Fisher Scientific) and PE-anti-CD25 (2.5 μl, cat 554866, BD Bioscience) and incubated on ice for 30 min. Cells were washed with DPBS with 2% BSA and fixed. To probe for intracellular transcription factors, cells were permeabilized according to the manufacturer’s protocol (Permeabilization Kit, cat 005523-00, eBioscience) and were reacted with APC-anti-Foxp3 (5 μl, cat 77-5775-40, eBioscience) for 1 h. Samples were analyzed using a BD LSRII flow cytometer and FACSDiva Software (BD Biosciences) at the UNMC Center Flow Cytometry Research Facility.

### Statistics

Data are presented as means ± SEM. Data were assessed for normal distribution by probability plots against the theoretical cumulative normal distribution function. Homoscedasticity of data was assessed by Cochran C, Hartley, Bartlett test, and Levene’s test. Normally distributed, homoscedastic data were evaluated by parametric one-way ANOVA followed by Newman-Keuls *post hoc* tests (Statistica, v13, TIBCO Software, Inc., Palo Alto, CA, United States). Measurements of Treg function were assessed by linear regression analyses as a function of Treg:Tresp ratios. Differences in multiple linear Treg functions were determined as significant differences in slope or intercepts (Prism, v7, GraphPad Software, Inc., San Diego, CA, United States).

### Cardiovascular Safety Study in Dogs

The *in vivo* studies pertaining to this portion of the project were performed at Charles River Laboratories, formerly WIL Research Laboratories. All animals were housed individually in clean stainless steel cages in an environmentally controlled room. The cages were elevated above stainless steel flush pans, which were cleaned daily. Animals were individually housed as to prevent cross contamination as part of the Latin-square cross over design. Dogs were maintained in accordance with the Guide for the Care and Use of Laboratory Animals (National Research Council, 2011). The facilities at WIL Research were fully accredited by the Association for Assessment and Accreditation of Laboratory Animal Care International (AAALAC International). Environmental controls were set to maintain a temperature of 71°F ± 5°F (22°C ± 3°C) and relative humidity of 50% ± 20%. Temperature and relative humidity were monitored continuously. Fluorescent lighting was set to provide illumination for a 12-h light/12-h dark photoperiod. Reverse osmosis-purified municipal water was available *ad libitum*.

Male Beagle dogs, 8–12 months of age and weighing 9.5 ± 038 kg, were previously implanted with radiotelemetry transmitters and used in a Latin square cross-over design for administration of LBT-3627 or vehicle (10 mM Tris + 267 mM glucose, pH 8.5). Radiotelemetry implantation was performed by Charles River Laboratories in naïve colonies of dogs following standard operating procedures. The telemetrized dogs were then maintained as a non-naïve colony for non-GLP CV safety monitoring with an adequate washout period between administrations of each test article. At the time of implantation, dogs were surgically fitted with telemetry transmitters [TL11M3-D70-PCTR, Data Sciences International (DSI), Minnesota, United States] into the abdominal cavity under anesthesia. The transmitters have a fluid-filled catheter (coated with an antithrombotic film to inhibit thrombus formation) with the tip filled with a patented gel for blood pressure collection, and 2 ECG leads surgically implanted emulating a Lead II configuration. All dogs were allowed to recover for at least 2 weeks from the implantation of the telemetry device before administration of the test articles. LBT-3627 was provided to WIL Research by Longevity Biotech, Inc. A total of 4 dogs were on study with each of the 4 dogs receiving each dose with at least a 3-day washout period in between any single dose. The test compound, LBT-3627, was administered via s.c. injection at 3 dose levels and a vehicle alone (0, 0.14, 0.6, and 1.4 mg/kg). These dose levels were allometrically scaled from the dose ranges that were observed to be effective to prevent neurodegeneration in a mouse model of PD (Olson et al., 2015). When scaled from dog, these doses would equate to approximately 0.5, 2, and 5 mg/kg in rats, which is comparable to the three doses used in the current neurodegenerative models (0.6, 2, and 6 mg/kg).

Following a single injection of the test compound or vehicle to the dog, a host of parameters were tracked by radiotelemetry. Clinical observations included detailed daily physical examinations (before and after dosing) and continual observation for the first 4 h after dosing. Dogs were also weighed before, after, and throughout the study. Baseline arterial blood pressure (systolic, diastolic, and mean), pulse pressure, heart rate, electrocardiographic (ECG) waveforms, and body temperature were collected continuously for 1 h prior to administration of vehicle or test compound. Following administration of vehicle or test compound, the same parameters were collected continuously for at least 24 h. Cardiovascular parameters and body temperature data were averaged for 4 dogs per group to appropriate time intervals for statistical analysis. Two way ANOVA was used to determine statistically significant differences between groups over time for each of the parameters that were tracked.

## Results

### Multiple Doses of VIPR2 Agonist Increase Treg Activity Without Expanding CD4^+^ T Cells or Tregs

To assess the effects of VIPR2 agonist on Treg numbers and function, we treated rats with 5 daily s.c. doses of LBT-3627 at 0.06, 0.2, 0.6, 2.0, or 6.0 mg/kg/dose or with PBS vehicle (0 mg/kg). The day after the last injection, T cell and Treg frequencies from peripheral blood and spleens were assessed. Compared to vehicle-treated controls, no significant differences were detected in percentages of (a) CD3^+^ T cells among lymphocytes, (b) CD4^+^ T cells among CD3^+^ T cells, CD25^hi^ T cells among CD3^+^CD4^+^ T cells, or CD25^hi^Foxp3^+^ Tregs among CD3^+^CD4^+^ T cells in either peripheral blood (*P* value ranges = 0.5807 – 0.9583) (Figure 1A) or spleen (*P* value ranges = 0.3477 – 0.9999) (Figure 1B). Spleen cells from individual animals were enriched for CD3^+^CD4^+^CD25^hi^ Tregs and assessed for suppressive activity toward Tresp. Flow cytometric analysis indicated those CD4^+^CD25^+^ T cells exhibited a Treg phenotype with greater than 90% expressing Foxp3 (Figure 1B). Compared to animals treated with vehicle, Treg activity was significantly increased in animals treated with LBT-3627 at 0.6. 2.0, and 6.0 mg/kg once daily for 5 days, but no effect was detected in rats treated with lower concentrations at 0.06 and 0.2 mg/kg (Figure 1C). We also assessed whether a single dose of VIPR2 agonist was sufficient to upregulate Treg activity. We found that one dose of LBT-3627 at levels required to upregulate Treg activity with multiple doses did not alter Treg activity compared to control (P_slope_ and P_intercept_ > 0.8702) (Figure 1D). These data together demonstrated that LBT-3627 elevates Treg function without increasing frequencies and that multiple doses of LBT-3627 are necessary to increase Treg activity. Those effective dosing paradigms were chosen for evaluation in the two neurodegenerative models.

### Dopaminergic Neurodegeneration and Reactive Microgliosis Are Induced by Human Wild Type α-Syn Overexpression

**FIGURE 2:**
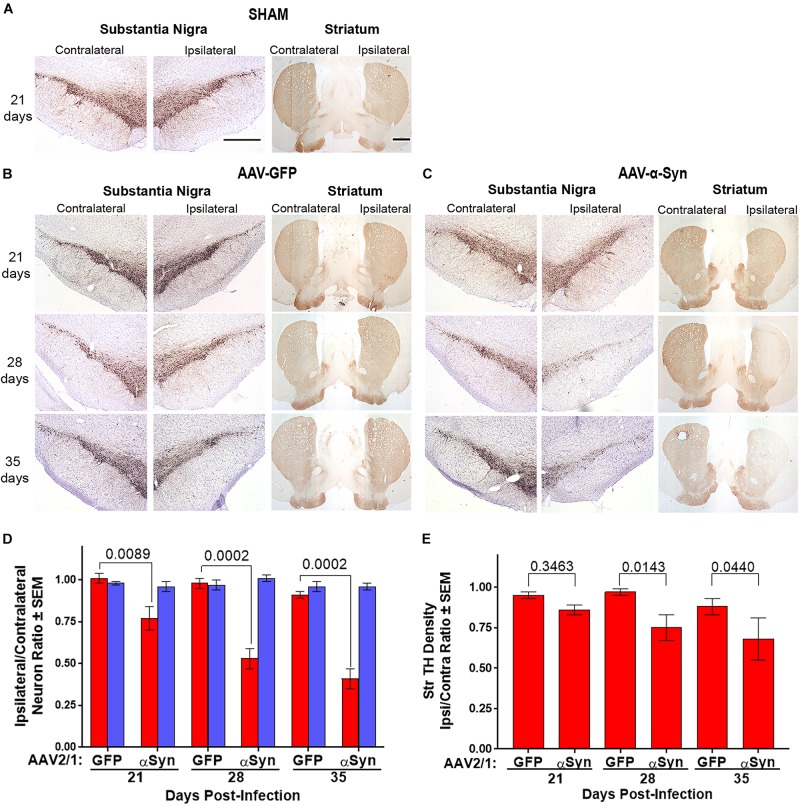
Dopaminergic neurodegeneration by overexpression of human α-Syn. Sprague-Dawley rats were stereotactically sham injected without infusion (*n* = 3/day) **(A)** or injected with either AAV-GFP vector (*n* = 5/day) **(B)** or AAV-α-Syn vector (*n* = 4–5/day) **(C)**. Brains were removed on days 21, 28, and 35 post injection. Sections of midbrain and striatum were stained to visualize tyrosine hydroxylase (TH) expression to reveal dopaminergic neurons in the substantia nigra and efferent termini in the striata. **(D)** Ipsilateral/contralateral ratios of TH^+^Nissl^+^ (red bars) and TH^+^Nissl^–^ (blue bars) neuron numbers as determined from stereological analysis. **(E)** Ipsilateral/contralateral ratios of TH^+^ striatal densities as determined from digital image analysis. Means and SEM were determined from 4 to 5 rats/group. Significant differences were assessed by one-way ANOVA and Newman-Keuls *post hoc* tests. *P*-values are denoted above connecting comparisons. Scale bars, 1000 μm.

We utilized the overexpression of human α-Syn in rat ventral midbrain as a model of dopaminergic neurodegeneration to assess putative neuroprotective modalities. Initially, to delineate the kinetics of neurodegeneration and inflammation in that model, Sprague-Dawley rats were stereotactically injected in the left hemisphere with AAV-α-Syn, AAV-GFP, or were sham-treated by insertion of the needle into the dura without infusion (SHAM). At 21, 28, and 35 days post-injection, animals were sacrificed, brains collected, processed, sectioned through the ventral midbrain and striatum, and stained for expression of TH for dopaminergic neurons and termini. TH staining was similar in sections isolated from rats treated with either sham (Figure 2A) or AAV-GFP (Figure 2B) in ipsilateral and contralateral hemispheres of the ventral midbrain and the striatum regardless of time after viral treatment indicating that viral delivery itself resulted in little if any neurodegenerative effect. In contrast, TH staining from animals treated with AAV-α-Syn showed reductions of TH staining within the substantia nigra and striatum of the ipsilateral hemisphere compared to the non-injected contralateral hemisphere (Figure 2C). These reductions are particularly evident 28 and 35 days after infection. To validate those observations, we counted TH^+^ and TH^–^ neuronal numbers in both the ipsilateral and contralateral hemispheres by stereological analysis and compared ratios of ipsilateral and contralateral neuronal counts. Significant loss of TH^+^Nissl^+^ neurons were not detected after treatment with AAV-GFP vector as ipsilateral/contralateral ratios at any time were greater than 0.91 and *P* > 0.3225 (Figure 2D). However, treatment with AAV-α-Syn resulted in 24, 36, and 55% loss of TH^+^Nissl^+^ neurons correlating to diminution of ipsilateral/contralateral ratios of 0.77, 0.53, and 0.41 on days 21, 28, and 35, respectively. No significant effects of treatment and/or day were detectable for non-dopaminergic (TH^–^Nissl^+^) neurons within the substantia nigra (*P* > 0.4603). While densities of striatal TH^+^ termini were slightly diminished 21 days after treatment with AAV-α-Syn, significant diminution was realized only after 28 and 35 days compared to treatment with AAV-GFP (Figure 2E).

**FIGURE 3:**
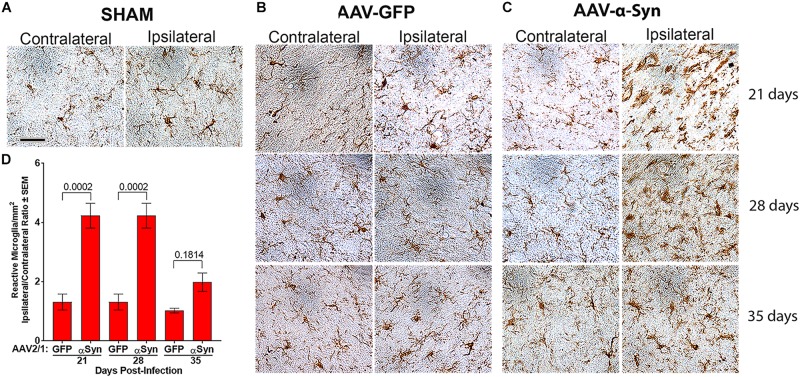
Human α-Syn overexpression increases reactive microglia. Sprague-Dawley rats (Figure 2) were stereotactically injected without infusion (SHAM) (*n* = 3/day) **(A)** or injected with either AAV-GFP vector (*n* = 5/day) **(B)** or AAV-α-Syn vector (*n* = 4–5/day) **(C)**. Brains were removed on days 21, 28, and 35 post injection. Sections of midbrain were stained to visualize Iba1 expression to reveal reactive microglia in the substantia nigra. **(D)** Numbers of Iba1^+^ amoeboid microglia were estimated by stereological analysis. Means and SEM were determined from 4 to 5 rats/group. Significant differences were assessed by one-way ANOVA and Newman-Keuls *post hoc* tests. *P*-values are denoted above connecting comparisons. Scale bar, 40 μm.

To assess the effects of α-Syn overexpression on neuroinflammation, sections of ventral midbrain were stained for Iba1 expression. Iba1^+^ microglia from sham (Figure 3A) and AAV-GFP (Figure 3B) treated rats showed mostly ramified morphologies with densities that were unaffected regardless of hemisphere, treatment regimen, or time after treatment. In contrast, by day 21 and through day 35 after AAV-α-Syn treatment, staining of amoeboid Iba1^+^ microglia were increased in the ipsilateral hemisphere compared to the contralateral hemisphere (Figure 3C). Stereological analysis confirmed these observations showing that ipsilateral/contralateral ratios of microglial densities (1.0 – 1.3) for AAV-GFP treated animals were not significantly different; however, AAV-α-Syn treatment increased those ratios to 4.2-fold by days 21 and 28 post-injection (Figure 3D). By day 35, the ratio diminished to twofold, which was significantly lower compared to those on day 21 and 28 (*P* < 0.0003), but not significantly different than AAV-GFP treated animals. These data suggested that by day 35, reactive microglia responses were beginning to be resolved, even in the context of progressive dopaminergic neuronal loss. Thus, we selected day 28 after AAV-α-Syn injection as an optimal time to assess the neuroprotective and anti-inflammatory capacities of VIPR2 agonist.

### VIPR2 Agonist Protects From Dopaminergic Neurodegeneration and Attenuates Neuroinflammation Induced by α-Syn Overexpression

**FIGURE 4:**
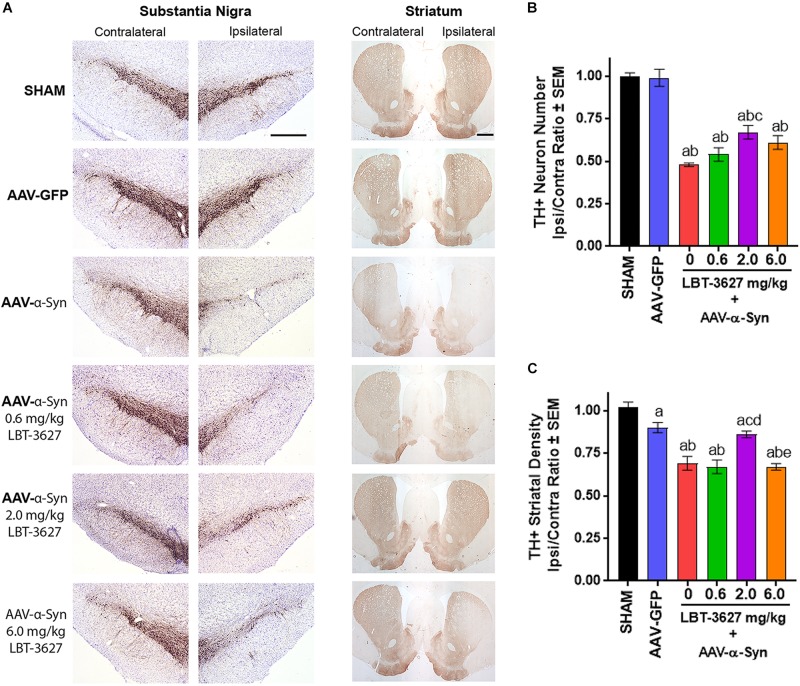
VIPR2 agonist protects dopaminergic neurons from α-Syn-mediated neurodegeneration. Sprague-Dawley rats were stereotactically injected, but not infused (SHAM) (*n* = 5) or infused with AAV-GFP (*n* = 6) or AAV-α-Syn vector (*n* = 6–7/group). AAV-α-Syn-treated rats began a regimen of PBS (0) (*n* = 7) or LBT-3627 at doses of 0.6, 2.0, or 6.0 mg/kg (*n* = 6/group) administered as daily s.c. injections for 5 days and every other day thereafter. On day 28 after infection, brains were obtained and sections of midbrain and striatum were stained for expression of TH **(A)**. **(B)** Numbers of TH^+^ neurons were assessed in the substantia nigra by stereological analysis and ipsilateral/contralateral ratios calculated. **(C)** Ipsilateral/contralateral ratios of TH^+^ densities were determined from digital image analysis of striatal TH expression. Means and SEM were determined from 5 to 7 rats/group. Significant differences were assessed by one-way ANOVA and Newman-Keuls *post hoc* tests. *P* ≤ 0.05 compared to treatment as ^a^sham controls, ^b^AAV-GFP, or animals treated with AAV-α-Syn and ^c^0 (vehicle), ^d^0.6, or ^e^2.0 mg/kg LBT-3627. Scale bars, 1000 μm.

To assess the effects of a VIPR2 agonist on neurodegeneration by α-Syn overexpression, we stereotactically injected rats with AAV-α-Syn and administered LBT-3627 in 5 sequential daily s.c. doses, followed by one dose every other day thereafter. Drug concentrations of 0, 0.6, 2.0, or 6.0 mg/kg/dose were delivered to each group. Control groups were treated with AAV-GFP or LBT-3627 vehicles. Assessment of brain sections from 28 days after AAV injections revealed that AAV-α-Syn led to reduced TH expression in the substantia nigra and striata compared to sham and AAV-GFP controls (Figure 4A). In contrast, intensities of TH expression within the ipsilateral hemispheres were increased in ipsilateral sections of the substantia nigra and striatum from AAV-α-Syn rats treated with LBT-3627 compared to those sections from vehicle-treated AAV-α-Syn rats. Stereological analysis validated findings within the nigra which demonstrated differences between the ipsilateral and contralateral hemispheres taken as ratios of TH^+^ neurons (Figure 4B). TH^+^ neuron ratios were significantly diminished in AAV-α-Syn treated animals compared to sham- and GFP-treated rats. While treatment with LBT-3627 after AAV-α-Syn injection increased TH^+^ neuron ratios, only 2.0 mg/kg significantly increased the ipsilateral/contralateral ratio by 43% compared to rats treated with AAV-α-Syn and vehicle. The increase ratio was due to increased numbers of dopaminergic neurons in the ipsilateral hemisphere compared to that of the AAV-α-Syn controls. Additionally, 2.0 mg/kg of LBT-3627 significantly spared the density of striatal TH^+^ termini by 24% compared to AAV-α-Syn controls (Figure 4C).

**FIGURE 5:**
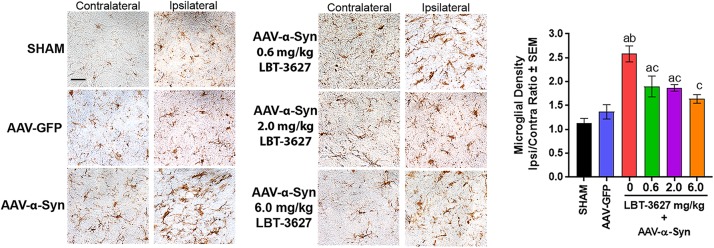
VIPR2 agonist diminishes microglial response to α-Syn overexpression. Sprague-Dawley rats were treated as sham control (*n* = 5) or stereotactically injected with AAV-GFP (*N* = 6) or AAV-α-Syn vector (*n* = 6–7/group). AAV-α-Syn-treated rats started the same regimen as in Figure 4 of vehicle (0) (*n* = 7) or LBT-3627 administered at 0.6, 2.0, or 6.0 mg/kg (*n* = 6/group). On day 28 after infection, brains were obtained and sections of midbrain were stained for expression of Iba1 by microglia. Numbers of Iba1^+^ amoeboid microglia were determined by stereological analysis. Means and SEM were determined from 5 to 7 rats/group. Significant differences were assessed by one-way ANOVA and Newman-Keuls *post hoc* tests. *P* ≤ 0.05 compared to treatments as ^a^sham controls, ^b^AAV-GFP, or AAV-α-Syn and ^c^PBS. Scale bar, 40 μm.

During the same study, we also assessed the effects of VIPR2 agonist on microglia-mediated neuroinflammatory responses. Sections of ventral midbrain from the above studies were probed for Iba1 expression and the densities of Iba1^+^ reactive microglia were measured. Sections showed that microglial morphologies and Iba1 intensities in ipsilateral and contralateral hemispheres were virtually identical 28 days after sham or AAV-GFP treatment (Figure 5). In contrast, α-Syn overexpression increased the intensity of staining for Iba1^+^ amoeboid microglia within the ipsilateral ventral midbrain compared to the contralateral hemisphere. LBT-3627 treatment diminished the intensity of Iba-1 staining in tissues from rats overexpressing α-Syn. To validate those observations, we enumerated reactive microglial by stereological analyses. AAV-α-Syn overexpression increased the numbers of ipsilateral Iba1^+^ reactive microglia by 2.5-fold compared to the contralateral hemisphere. Treatment with LBT-3627 at doses of 0.6, 2.0, and 6.0 mg/kg reduced reactive microglia by 26, 27, and 36%, respectively. Interestingly, doses of 6.0 mg/kg returned microglia numbers to levels not significantly different from either sham or AAV-GFP controls, resulting in nearly complete ablation of the microglia response.

### VIPR2 Agonist Rescues Treg Activity Diminished by α-Syn Overexpression

**FIGURE 6:**
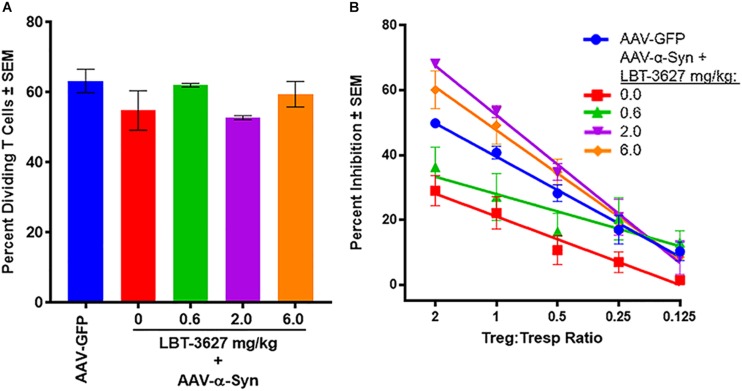
VIPR2 augments Treg activity in rats overexpressing human α-Syn. Sprague-Dawley rats were treated by stereotaxic injection with AAV-GFP (*n* = 3) or AAV-α-Syn vector. Rats treated with AAV-α-Syn-began a regimen of LBT-3627 administrated at 0.6, 2.0, or 6.0 mg/kg or vehicle (0 mg/kg) (*n* = 3/group) by daily administration for 5 days and every other day thereafter. On day 28 after infection, spleen cells were enriched for conventional CD4^+^ T cells and assessed for percentage of proliferating T cells **(A)**, or enriched for Tregs and assessed for percentage inhibition of proliferating T cells **(B)**. Note, treatment regimens for **(A,B)** are color-matched. **(A)** Means and SEM were determined for 3 rats/group. No significant differences were detected by one way ANOVA, *P* = 0.2028. **(B)** Significant differences of Treg activities between treatment groups were determined from linear regression analysis of 3 rats/group. Regression analysis for all treatments indicated that each regression exhibited *R*^2^ > 0.83 with *P* values < 0.032.

As Treg function has been shown to be deficient in both PD patients and in mice immunized with α-Syn, and since LBT-3627 augments Treg activity with subsequent neuroprotection (Reynolds et al., 2010; Saunders et al., 2012; Olson et al., 2015), we assessed the effects of VIPR2 activation on Treg numbers and function in the context of α-Syn overexpression. As described above, rats were stereotactically injected with AAV-α-Syn and treated with varying doses of LBT-36327. After 28 days, splenic cells were enriched for Tresp and Treg populations and tested for proliferative or suppressive functions, respectively. Tresps were stained with CFSE, stimulated with CD3/CD28 beads in the absence of Tregs, and assessed by flow cytometric analysis for proliferative capacity after 3 days of incubation. No significant differences in the percentages of proliferating Tresps were discernable between treatment groups (*P* = 0.2028) (Figure 6A). In contrast, Treg function from AAV-α-Syn-treated rats (red line) was significantly diminished compared to Treg function from AAV-GFP controls (blue line) (*P* = 0.0115) (Figure 6B). Treatment with 0.6 mg/kg of LBT-3627 (green line) increased Treg function compared to that from AAV-α-Syn alone treated rats (*P* = 0.0065), but was still lower than that from AAV-GFP controls (blue line) (*P* = 0.0168). Only doses of 2.0 (purple) and 6.0 (orange) mg/kg of LBT-3627 were sufficient to increase Treg activity above that of GFP controls (*P* < 0.007). While no significant differences in Treg function were detected between 2.0 and 6.0 mg/kg doses, 2.0 mg/kg doses of LBT-3627 tended to show better efficacy affecting Treg function and TH^+^ neuron survival.

### Higher Doses of VIPR2 Agonist Are Required to Protect Dopaminergic Neurons in the 6-OHDA Model

**FIGURE 7:**
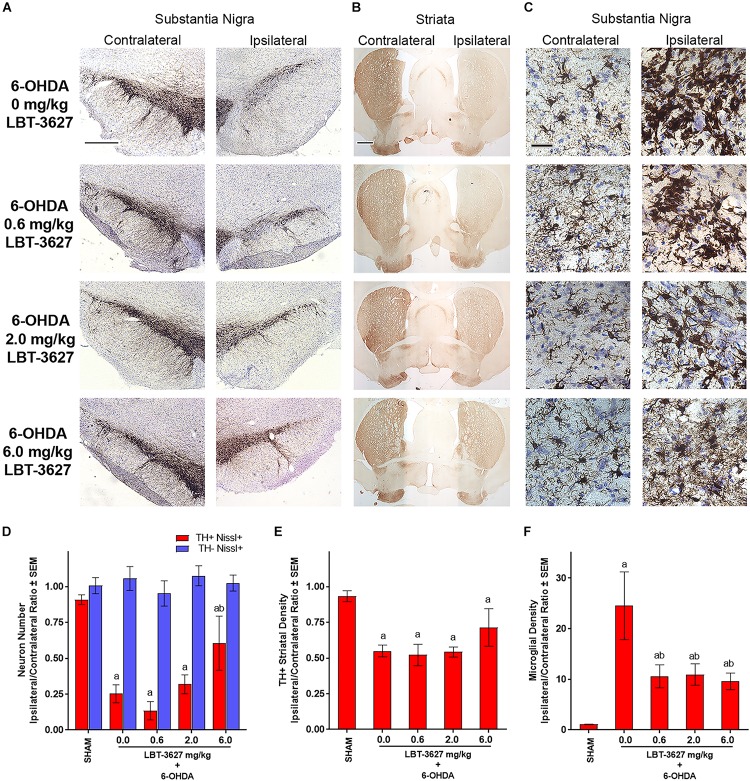
VIPR2 agonist protects dopaminergic neurons and diminishes microglial response after 6-OHDA. Lewis rats were administered 10 μg of 6-OHDA in 5 μl of PBS by stereotaxic injection or were injected without infusion (SHAM controls) (*n* = 6). Immediately after injection, rats began a regimen of either vehicle (0.0 mg/kg) (*n* = 5) or 0.6 (*n* = 6), 2.0 (*n* = 6), or 6.0 (*n* = 5) mg/kg of LBT-3627 administered as daily injections for 5 days and every other day thereafter. After 14 days, brains were obtained, processed and stained for expression of TH in the substantia nigra **(A)** and striatum **(B)**, and for Iba1 in the substantia nigra **(C)**. **(D)** TH^+^Nissl^+^ (red bars) and TH^–^Nissl^+^ (blue bars) neurons within the substantia nigra were counted by stereological analysis and the ipsilateral/contralateral ratios of those numbers were determined. No significant differences in TH^–^Nissl^+^ neurons were discernible between treatment groups, *P* = 0.7671. **(E)** Densities of TH^+^ termini were determined by digital image analysis and ipsilateral/contralateral ratios of those densities calculated. **(F)** Densities of amoeboid Iba1^+^ microglia were assessed by stereological analysis and ipsilateral/contralateral ratios of those densities were determined. **(D–F)** Means and SEM were calculated for 5–6 rats/group. Significant differences were assessed by one-way ANOVA followed by Newman-Keuls *post hoc* tests. *P* ≤ 0.05 compared to animals treated as ^a^sham controls or with ^b^6-OHDA + vehicle (0 mg/kg). Scale bars, **(A,B)** 1000 μm and **(C)** 40 μm.

We sought to validate the neuroprotective capacity of VIPR2 agonist in a second model of dopaminergic neurodegeneration. For these studies, 6-OHDA was delivered to the medial forebrain bundle by stereotactic injection into the left hemisphere while the contralateral hemisphere served as a control. Following a similar dosing paradigm as described above, immediately after 6-OHDA delivery, LBT-3627 at doses of 0.6, 2.0, or 6.0 mg/kg was initiated with 5 daily s.c. injections followed every other day thereafter until day 21 post-injection. A control group included rats that received 6-OHDA and were treated with vehicle alone. Sections of midbrain and striatum immunostained for TH expression showed substantive loss of TH expression within the substantia nigra of the ipsilateral hemisphere compared to the contralateral side (Figure 7A). Meanwhile, examination of sections from animals treated with LBT-3627 suggested that ipsilateral expression of TH in the substantia nigra was increased in a dose-dependent fashion compared to 6-OHDA controls. TH^+^ stained termini in the striatum indicated that 6-OHDA resulted in a profound loss of TH expression in the ipsilateral hemisphere compared to contralateral hemisphere (Figure 7B). Additionally, sections stained for Iba1^+^ reactive amoeboid microglia in the ventral midbrain, revealed that 6-OHDA treatment induced an intense activated microglial response (Figure 7C). Treatment with LBT-3627 at all doses substantially decreased neuroinflammatory responses as exhibited by diminished Iba-1 expression intensity. Stereological analysis of TH^+^Nissl^+^ neurons within the ipsilateral substantia nigra revealed a 73% loss of dopaminergic neurons by 6-OHDA compared to sham control (Figure 7D). Treatment with 6.0 mg/kg of LBT-3627 spared 53% of the dopaminergic neurons, whereas doses of 0.6 and 2.0 mg/kg showed no significant effect compared to 6-OHDA control. No effect on TH^–^Nissl^+^ non-dopaminergic neurons was detected (*P* = 0.7671). Digital image analysis of TH^+^ striatal termini showed a 41% loss of dopaminergic termini compared to sham controls, and regardless of LBT-3627 dose, losses of striatal termini were not significantly different from 6-OHDA controls (*P* > 0.1226) (Figure 7E). Meanwhile, to measure the inflammatory response of 6-OHDA, the density of Iba1^+^ reactive amoeboid microglia were determined by stereological analysis. 6-OHDA induced a 22.6-fold increase in the density of reactive microglia in the ipsilateral hemisphere compared to that of the sham control (Figure 7F). Treatment with LBT-3627 decreased reactive microglia densities in the SN by 57–61%, regardless of dose. Taken together these data indicate that LBT-3627 is neuroprotective at the 6 mg/kg dose in 6-OHDA-induced dopaminergic neurodegeneration, while attenuation of microglial-mediated neuroinflammatory responses was possible at all dose levels evaluated.

### LBT-3627 Tests on Cardiovascular Metrics

**FIGURE 8:**
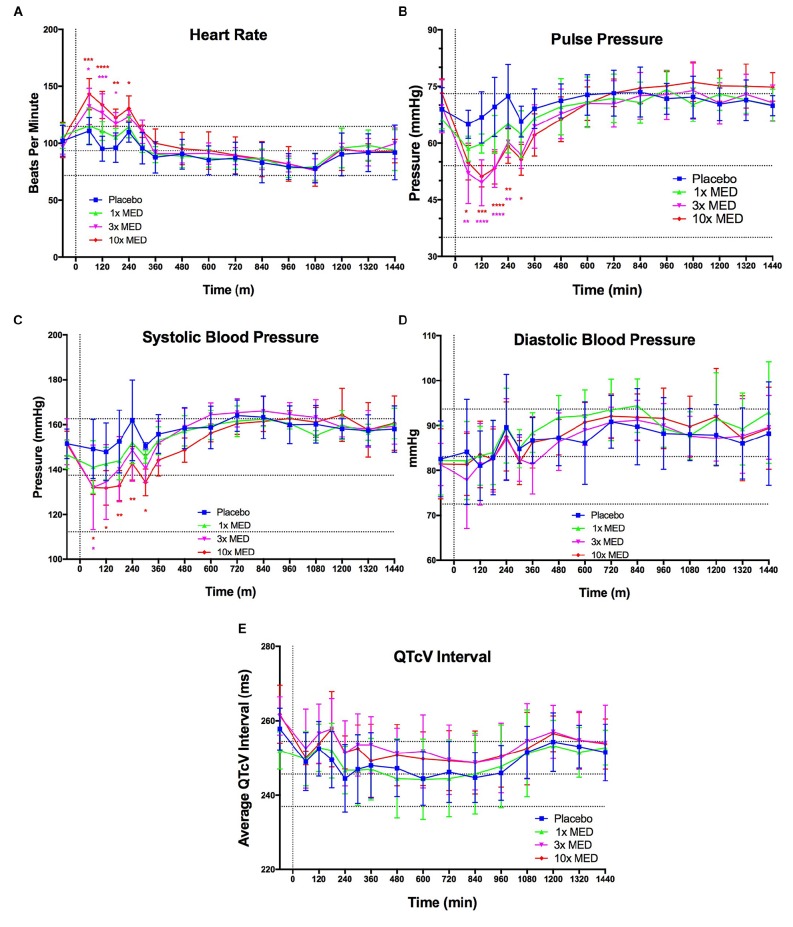
VIPR2 agonist does not induce any overtly concerning effects on CV parameters. LBT-3627 was administered subcutaneously to beagle dogs at three dose levels (1× minimum effective dose (MED) = 0.14 mg/kg, 3× MED = 0.6 mg/kg, and 10× MED = 1.4 mg/kg), plus a vehicle only control (placebo), that allometrically scale to approximately match the three dose levels used in the neuroprotection portion of this study (0.6, 2.0, and 6.0 mg/kg) (*n* = 4/group). CV parameters were tracked for 24 h after the single s.c. injection. Additionally, horizontal dotted lines indicate the historic baseline averages, plus and minus one standard deviation, for the dogs within the testing colony. For the lowest dose level (1× MED), none of the CV parameters were significantly modulated compared to placebo **(A–E)**. Heart rate was significantly increased for both 3× and 10× MED for the first 3–4 h after administration and returned to placebo levels after 4 h **(A)**. Pulse pressure mirrors this response and is increased for the 3× and 10× MED groups for the first 4 h **(B)**. This increase in pulse pressure can be attributed to the increase in systolic pressure **(C)**, since the diastolic pressure was not modulated for any dose level compared to placebo **(D)**. QTcV interval was unaltered for any (dose level compared to placebo as well **(E)**. Means and standard deviations were calculated for 4 dogs/group and significant differences were assessed by two-way ANOVA with Dunnett’s *post hoc* tests. Compared to placebo treated animals, designated *P* values were ^∗^≤0.05, ^∗∗^≤0.01, ^∗∗∗^≤0.001, or ^****^≤0.0001.)

Despite promising immunomodulatory and related neuroprotective effects of VIPR2 activation observed in both rat models investigated in this report, a major gap between preclinical and human efficacy existed. To advance this VIPR2 strategy toward clinical evaluations warranted safety evaluations of LBT-3627. Thus, we administered LBT-3627 by subcutaneous (s.c.) injection to dogs at doses of 0.14, 0.6, and 1.4 mg/kg, which were allometrically scaled to mirror dose concentrations required for Treg induction. After the single injection of LBT-3627, dogs were monitored telemetrically for heart rate, pulse pressure, systolic blood pressure, diastolic blood pressure, and corrected Van de Water’s QT (QTcV) interval (Figure 8). At the lowest dose level, none of the monitored parameters were changed over placebo during a 24-h observation period. At the two higher doses, transient, but limited, increases in heart rate, decreases in pulse pressure, and reductions in systolic pressure were observed. All changes were within the historical range of baselines as indicated by the dotted horizontal boundaries in all plots (Figures 8A–E).

## Discussion

The study provides further evidence that modulation of adaptive immunity through the production of Tregs leads to significant neuroprotection in PD animal models. As a peptide hormone and neurotransmitter, VIP’s pleotropic effects modulate innate and adaptive immunity (Delgado et al., 2004; Pozo and Delgado, 2004; Reynolds et al., 2010; Ganea et al., 2015; Olson et al., 2015) that include the inhibition of macrophage and microglial release of inflammatory mediators (Gonzalez-Rey and Delgado, 2005, 2008; Delgado et al., 2008; Waschek, 2013; Higyno et al., 2015), regulation of lymphocytic Th1/Th2/Th17 differentiation (Voice et al., 2004; Jimeno et al., 2012; Tan et al., 2015; Villanueva-Romero et al., 2018), and alterations of immunoglobulin production by B lymphocytes (Samarasinghe et al., 2011; Xu et al., 2014). Our prior works also show that VIP and selective VIPR1 and VIPR2 receptor analogs affect Treg activity (Reynolds et al., 2010; Olson et al., 2015, 2016). In the setting of neurodegenerative diseases, VIP and VIP-based analogs downregulate microglia pro-inflammatory activities and spare dopaminergic neuronal damage in the nigra and striatum. These activities appear to be Treg-mediated as adoptive transfer from VIP-treated donors with α-Syn-specific Th17 effectors resulted in dopaminergic neuroprotection (Reynolds et al., 2010). Parallel experiments wherein Tregs were transferred from naïve donors were not as protective. In each of these cases, Treg-mediated activities induced by VIPR2 agonist performed better than those induced by VIPR1 agonist or native VIP (Olson et al., 2015). All together, these data show the importance of the VIPR2 receptor, not only as an immune modulator, but also as a potentiator of Treg-mediated neuroprotection (Dejda et al., 2005; Rangon et al., 2005; Delgado et al., 2008; Reynolds et al., 2010; Shioda and Gozes, 2011; Olson et al., 2015, 2016; Deng and Jin, 2017).

In parallel, VIP is associated with adverse effects in the gastrointestinal and the CNS (Bloom et al., 1973; Said and Faloona, 1975; Bloom, 1978; Kane et al., 1983; Guelrud et al., 1992; Masel et al., 2000; Cernuda-Morollon et al., 2015; Riesco et al., 2017). VIP’s effects are due in large part by engaged signaling pathways associated with binding of specific receptors preferentially expressed by specific cell types. For instance, VIPR1 is preferentially expressed by gastrointestinal tissues, while VIPR2 is relegated largely to smooth muscle, lung, and myocardial organs (Schulz et al., 2015; Jayawardena et al., 2017). In the immune system, early reports suggested that modulatory effects were associated with the constitutively expressed VIPR1, however, recent data suggest that induced VIPR2 may play a larger role than previously suggested, especially for Treg induction or potentiation of suppressive function (Goetzl et al., 2001; Martinez et al., 2002; Samarasinghe et al., 2011; Olson et al., 2015, 2016). We recently synthesized specific agonists specific for VIPR1 or VIPR2 that do not interact with ADCYAP1R1 (PAC1), and display increased metabolic stability compared to VIP (Olson et al., 2015). We demonstrated that pretreatment with the VIPR2-specific agonist, LBT-3627, but not a VIPR1 agonist (LBT-3393), potentiated Treg neuroprotective activity in a dose-dependent manner in MPTP mice. These results supported Treg induction experiments in VIPR knockout animals which concluded that VIPR2, rather than VIPR1 plays a greater role in VIP-mediated augmentation of Tregs (Yadav et al., 2011; Tan et al., 2015). Moreover, adoptive transfer studies demonstrated that cells from LBT-3627-treated animals provide significantly greater protection for dopaminergic neurons in MPTP mice than that provided by VIPR1 agonist (Olson et al., 2015, 2016). However, whether VIPR2 agonist functions in different species and affords neuroprotection in more progressive models of dopaminergic neurodegeneration awaits further investigation.

In establishing the neuroprotective profile of VIPR2, adoptive transfer techniques were necessary to overcome immunotoxicities intrinsic to the MPTP model. Herein, we used two different animal models that are not inherently immunotoxic and, therefore, preclude the need for adoptive transfer to evade toxicities and elicit neuroprotection. The α-Syn overexpression model results from this study are particularly interesting as they establish a clear link between human PD pathology (accumulation of α-Syn in the brain) and immune system dysregulation of innate and Treg functions (Figures 3, 6). Of particular interest is that each dose level of LBT-3627 tested reduced microglial activation regardless of dose. Most interestingly, the middle dose of LBT-3627 (2.0 mg/kg), which trended to provide the highest suppressive activity, paralleled neuroprotection. Similar observations were recorded in the 6-OHDA rat model, wherein LBT-3627 reduced microglial activation across all three dose levels, but only the highest dose (6.0 mg/kg) was sufficient to afford significant neuronal survival. While the exact biological cascade fully describing how VIPR2 activation in the periphery leads to central neuroprotection remains incomplete, it is clear that peripheral T cell function plays a critical role. Several groups, ours included, are investigating specific genomic pathways outlining the crosstalk between CNS and immune compartments (Reynolds et al., 2010; Ransohoff and Brown, 2012; Kosloski et al., 2013; Anderson et al., 2014; Olson et al., 2015; Chan et al., 2016; Gendelman et al., 2017; Schutt et al., 2018; Limanaqi et al., 2019). Other groups have reported that the parenchyma and/or choroid plexus contribute to the crosstalk, which becomes more pronounced in diseased states, while others have sidestepped the issue and allude to passive diffusion pathways (Xu et al., 2010; Nunan et al., 2014; Balusu et al., 2016; Jayawardena et al., 2017; Marques et al., 2017; Arcuri et al., 2019).

However, should subsequent work determine that VIPR2 agonist is indeed directly responsible for both peripheral and central observations noted herein, without additional messengers, the neurotrophic aspects of the VIPR2 receptor would come into play. Specifically, VIP interneurons play several key roles in cognition. Recent work has revealed that VIP interneurons are able to affect the function of downstream pyramidal neurons. In optogenetic animal models, activation of these VIP interneurons improved memory retention over negative controls (Kamigaki and Dan, 2017). Furthermore, activation or disinhibition of these neurons, releases a self-imposed suppression on interneuron neurotransmitter signaling (Lee et al., 2013; Pi et al., 2013; Fu et al., 2014; Stryker, 2014; Zhang et al., 2014). Perhaps an underlying cause for this biological cascade affects circadian rhythm aspects relevant to VIP biology (Ono et al., 2016).

Aside from the biology described above, VIP is also responsible for attenuating microgliosis (Kim et al., 2000; Dejda et al., 2005; Waschek, 2013). This orthogonal, yet direct, mechanism of action is also beneficial in the context of a neurodegenerative disorder. If the complex biological pathways and peripheral to central crosstalk thesis is ignored as “too complex,” one must conclude that the VIPR2 agonist is a lone actor with central actions being all neuroprotective in the diseased state and ultimately resulting in improved memory recall. While much work remains to be done to understand the peripheral to central transduction pathway along with a multitude of downstream molecular actors involved, the results herein clearly indicate that something is transferring from the peripheral to central compartments resulting in robust neuroprotection with attenuation of microgliosis. Therefore, based on our collective work, we proposed that VIPR2 activation, though minimally on Tregs, but potentially on other cell types, initiates a cascade of biological events that include (1) improved peripheral Treg function, (2) reduced reactive microglia inside the central compartment, (3) shifted balance of multiple pro- and anti-inflammatory cytokines, and (4) robust, dose response-mediated neuroprotection of TH^+^ dopaminergic neurons.

Interestingly, an optimal range in the VIPR2 agonist dose appears to be necessary for Treg functional improvement in terms of neuroprotection. Specifically, in our earlier work, high concentrations of VIPR2 agonists result in a decrease in cAMP production via cell-based assays (Olson et al., 2015). This has been attributed to GPCR activation-induced receptor internalization (Valdehita et al., 2010). The rescue of Treg function in the α-Syn model was evident at the same dose (2 mg/kg) that induced the greatest neuroprotection. On the other hand, neuroprotection in the 6-OHDA model was attained only at the highest dose level. The case may be made that an appropriate balance must be achieved under different disease processes and conditions, and that a standard dose level may not be the best for each different set of circumstances. Another explanation to the non-traditional dose-response outcomes for neuroprotection in the α-Syn model may lie within the complex GPCR biological cascades that act through cAMP, β-arrestin1 or β-arrestin2 as second messengers. Furthermore, these underlying circuits may be wired differently for different cell types, possibly explaining the nuanced results reported herein. Multi-omic approaches would shed additional light on the specific wiring of this therapeutic pathway.

Regardless of the disease model, all dosing regimens of LBT-3627 reduced numbers of inflammatory microglia. For the overexpression of α-Syn model, treatment with LBT-3627 caused a dose-dependent reduction in microglial activation with 6 mg/kg yielding microglia levels indiscernible from sham or GFP controls, albeit not yielding statistically different levels from the other two lower doses. Similarly, LBT-3627 in 6-OHDA treated animals reduced microglia equally, regardless of dose, but did not attain levels of sham controls. This dichotomy may be due, in part, to different intensities of microglial responses in each model. Numbers of inflammatory microglia in 6-OHDA-treated animals increased 24-fold compared to sham controls, whereas overexpression of α-Syn increased microglial numbers by only 2.6-fold compared to sham controls. Thus, microglial responses in 6-OHDA treated rats intensities were 10-fold greater than those in α-Syn overexpression animals. The intensity of the microglial response may also affect the adaptive immune responses and exacerbate neuroinflammation and neurodegeneration as previously reported and reviewed (Benner et al., 2008; Gendelman and Mosley, 2015; Mosley and Gendelman, 2017). Indeed, 6-OHDA treated mice also have been reported to exhibit an extensive microglial response associated with IgG deposition, increased infiltration of T and B cells, and exacerbated dopaminergic neurodegeneration (Theodore and Maragos, 2015). Moreover, α-Syn overexpression induced an average of 50% dopaminergic neuronal loss in the substantia nigra, whereas 6-OHDA induced a 75% loss. Together, these data suggest that 6-OHDA induces greater severity of neuroinflammation and neurodegeneration than α-Syn overexpression.

It is well-known that peripheral VIPR2 activation can be directly attributed to central neuroprotection along with a reduction in microgliosis. Using doses of 2 mg/kg LBT-3627 led to protection of dopaminergic neurons in the SNpc and their terminal processes in the striatum reflecting our own prior works in MPTP intoxicated animals (Olson et al., 2015). Notably, doses of 2 mg/kg nearly restored striatal density to levels of AAV-GFP controls (Figure 4C). Nonetheless, in 6-OHDA-treated rats, doses of 2 mg/kg LBT-3627 were insufficient to elicit neuroprotection along the nigrostriatal axis and required at least 6 mg/kg to protect nigral neurons. Alternatively, differences in the exhibited effective doses for neuroprotection between models may be influenced by the difference in the severity of the models. Thus, regarding translational evaluation, the therapeutic paradigm may require personalization by dose titration or a biomarker guided treatment strategy using T cell-related markers as pharmacodynamic surrogates. For example, Treg activity may be one such marker since our works confirmed that VIPR2 agonism enhanced Treg activity, which confirmed others’ previous results as well that required multiple injections to augment Treg activity in healthy subjects (Delgado et al., 2005; Chen et al., 2008; Deng et al., 2010; Jimeno et al., 2010; Reynolds et al., 2010; Olson et al., 2015, 2016).

While VIP and VIPR2 agonism promote Treg induction and function, the mechanisms are not well-defined. VIPR2 expression is either absent or minimal on resting CD4^+^ T cells and Tregs, but can be upregulated by activated CD4^+^ T cells (Lara-Marquez et al., 2001). Additionally, Tregs from lymphoid tissues of VIPR2 deficient mice are found at lower levels and have a diminished capacity to expand after stimulation, suggesting a role for VIPR2 in VIP- or agonist-induced maintenance or potentiation of Treg numbers or function. Whether that role is limited to direct interactions by Tregs or can indirectly exert its effects through other cell types has yet to be definitively resolved. Indeed, we previously showed that the VIPR2 agonist, LBT-3627 induces a 45-fold increase in GM-CSF expression among CD4^+^ T cells (Olson et al., 2015), and that GM-CSF-induced tolerogenic DCs to transform naïve CD4^+^ T cells to Tregs by OX40L and Jagged1 induced signaling via OX40 and Notch1, respectively (Gopisetty et al., 2013; Alharshawi et al., 2017; Schutt et al., 2018). Underscoring the translational potential of VIPR2 agonism is suggested by the demonstration that those downstream signaling outcomes via GM-CSF have successfully been tested in clinical trials of PD wherein sargramostim-treated patients showed increased Treg numbers and function, augmented motor neuronal activity, and improved UPDRS III scores (Gendelman et al., 2017). A direct mechanism is suggested for T cells activated through CD3/CD28, whereby VIP induced cell cycle arrest, inhibited IL-2 transcription, and suppressed transcription factor-mediated signaling with eventual transformation of Teff phenotypes to Treg phenotypes with high levels of CD25, CTLA4, and FOXP3 expression as well as potent suppressive activities (Anderson and Gonzalez-Rey, 2010). Indeed, the first translational use of VIP for regulation of immune conditions was tested with inhaled VIP for inflammatory sarcoidosis which increased Treg numbers and function from T cells in bronchoalveolar lavages (Prasse et al., 2010). Together, these data provide strength for a therapeutic strategy of VIP and VIP agonists as immune modulatory agents in inflammatory- or immune-mediated conditions.

Finally, we and others have shown that specific T cell responses are associated with PD (Baba et al., 2005; Brochard et al., 2009; Saunders et al., 2012; Levite, 2016; Gendelman et al., 2017; Mosley and Gendelman, 2017; Sulzer et al., 2017; Kustrimovic et al., 2018). Previous clinical studies showed diminution of Treg numbers or activities in PD patients compared to non-PD controls whereby those diminutions correlated to disorder severity (Saunders et al., 2012; Gendelman et al., 2017; Kustrimovic et al., 2018). Moreover, exposure to modified α-Syn has been shown to diminish Treg activity and Treg numbers (Reynolds et al., 2008, 2009, 2010; Labrador-Garrido et al., 2014; Schutt et al., 2018). Herein, we also demonstrated that Treg activity was diminished in rats that overexpress α-Syn compared to GFP controls (Figure 6), but showed no untoward effect on numbers or proliferative function of conventional CD4^+^ T cells that serve as Tresps. Treatment of α-Syn overexpressing rats with LBT-3627 at 0.6 mg/kg slightly increased Treg activity from the nadir attained in α-Syn expressing rats, but not to Treg levels of controls. Rescue of Treg activity above control levels required treatment with VIPR2 agonist concentrations of 2–6 mg/kg. These data confirmed our previous data which demonstrated that immunization with nitrated α-Syn diminished Treg activity to levels significantly below naïve controls even with concomitant increases in Treg numbers (Reynolds et al., 2010). More recently, we found that nitrated α-Syn added during *in vitro* differentiation of naïve T cells to Tregs by tolerogenic dendritic cells significantly diminished numbers of differentiated Tregs (Schutt et al., 2018). In previous clinical studies, we found that Treg function levels were significantly diminished in PD patients compared to age- and environment-matched controls (Saunders et al., 2012; Gendelman et al., 2017). Similar to results here, we also found that treatment with VIP at the time of immunization with α-Syn rescued deficient Treg activity to levels significantly above controls (Reynolds et al., 2010). More importantly, treatment of PD patients or MPTP-intoxicated mice with another immune modulatory agent, GM-CSF, also rescued Treg function and improved clinical scores as well as motor function (Kosloski et al., 2013; Gendelman et al., 2017). Taken together, these data indicate that that modified α-Syn regardless of endogenous or exogenous derivation, interferes with Treg differentiation or activation and diminishes Treg capability to control neuroinflammation. Thus, VIP or VIPR2 agonist may serve as a putative therapeutic to rescue deficits of differentiation and activation and return Treg function to neuroprotective levels.

## Data Availability

Data supporting the conclusions of this manuscript will be made available by the authors, without undue reservation, to any qualified researcher.

## Ethics Statement

The animal study was reviewed and approved by the UNMC Institutional Animal Care and Use Committee and Charles River Laboratories.

## Author Contributions

RM, JS, SS, and HG designed the experiments, interpreted the results, and wrote the manuscript. JS and SS provided the LBT-3627. YL, KO, JM, WY, and KN performed the experiments. YL and KO acquired and analyzed the data. RM performed the statistical analysis. All authors read and approved the final manuscript.

## Conflict of Interest Statement

JS and SS are employees and shareholders of the Longevity Biotech, Inc. The remaining authors declare that the research was conducted in the absence of any commercial or financial relationships that could be construed as a potential conflict of interest.
